# Pedophilic Disorder and Personality Pathology in German Forensic Psychiatry Institution: Geographical and Diagnostic Correlations

**DOI:** 10.1192/j.eurpsy.2026.10950

**Published:** 2026-07-31

**Authors:** J. Eberlein, K. Manaridou, C. Dokos

**Affiliations:** 1 St. Josef Psychiatric Hospital AMEOS, Oberhausen; 2 LWL-Center for Forensic Psychiatry, Lippstadt; 3 Department of Acute and Emergency Medicine, Klinikum Luedenscheid, Luedenscheid, Germany

## Abstract

**Introduction:**

Personality disorders (PD) are among the most prevalent psychiatric conditions in forensic populations. Systematic reviews indicate that up to two-thirds of male prisoners are affected, with antisocial traits being particularly widespread (Fazel S, Danesh J. Lancet 2002; 359:545–550). Borderline personality disorder (BPD) is also commonly found in incarcerated groups and frequently co-occurs with other cluster B features (Looper KJ, Paris J. Compr Psychiatry 2000; 41:432–437). Notably, PD pathology often overlaps with paraphilic disorders, where associations with antisocial and BPD traits have been demonstrated (Eher R, et al. Acta Psychiatr Scand 2019; 139:572–581). These comorbidities may contribute to offending behavior and thus heighten the risk of recidivism. Despite the clinical and forensic relevance, systematic data for German forensic institutions remain limited.

**Objectives:**

To examine geographical distribution, anthropometric factors, and especially diagnostic correlations in patients diagnosed with pedophilic disorder.

**Methods:**

Data were extracted retrospectively from the records of 30 male patients admitted to the LWL-Center for Forensic Psychiatry in Lippstadt, Germany. Diagnoses were made according to DSM-IV and included pedophilic disorder (F65.4), combined or unspecified PD (F61), BPD (F60.31), and mild intellectual disability (F70.1). Additional variables comprised age, nationality, residential location, and anthropometric measurements. Statistical analyses were conducted with SPSS 16.0, and a multilayer perceptron (MLP) neural network was applied to detect diagnostic correlations and clustering patterns. The study was conducted in accordance with institutional and ethical standards.

**Results:**

The mean age of the patients was 44.2±13 years. All participants were German nationals with residences broadly distributed across North Rhine-Westphalia. The most frequent diagnoses were F65.4, F61, F60.31, and F70.1. Cluster and MLP analyses revealed a notable association between pedophilic disorder (F65.4) and combined/other PD (F61), along with an additional link to BDP (F60.31). Anthropometric variables did not form relevant clusters, and geographical mapping showed no regional hotspots.

**Conclusions:**

These findings highlight the central role of pedophilic disorder in forensic psychiatry, particularly its frequent co-occurrence with PD pathology (F61) and BDP traits (F60.31). The application of MLP allowed hidden diagnostic relationships to be uncovered and may serve as a promising methodological tool for future psychiatric epidemiology.

**Disclosure of Interest:**

None Declared

